# Metabolomic Analysis of Response to Nitrogen-Limiting Conditions in *Yarrowia* spp.

**DOI:** 10.3390/metabo11010016

**Published:** 2020-12-29

**Authors:** Sivamoke Dissook, Sastia Prama Putri, Eiichiro Fukusaki

**Affiliations:** Department of Biotechnology, Graduate School of Engineering, Osaka University, 2-1 Yamadaoka, Suita, Osaka 565-0871, Japan; sivamoke@bio.eng.osaka-u.ac.jp (S.D.); fukusaki@bio.eng.osaka-u.ac.jp (E.F.)

**Keywords:** *Yarrowia*, LC-MS/MS, metabolome

## Abstract

*Yarrowia* is a yeast genus that has been used as a model oleaginous taxon for a wide array of studies. However, information regarding metabolite changes within *Yarrowia* spp. under different environmental conditions is still limited. Among various factors affecting *Yarrowia* metabolism, nitrogen-limiting conditions have a profound effect on the metabolic state of yeast. In this study, a time-course LC-MS/MS-based metabolome analysis of *Y. lipolytica* was performed to determine the optimal cultivation time and carbon-to-nitrogen ratio for studying the effects of nitrogen-limiting conditions on *Yarrowia*; we found that cultivation time of 36 h and carbon-to-nitrogen ratio of 4:1 and 5:0 was suitable for studying the effects of nitrogen-limiting conditions on *Yarrowia* and these conditions were applied to six strains of *Yarrowia*. These six strains of *Yarrowia* showed similar responses to nitrogen-limiting conditions; however, each strain had a unique metabolomic profile. Purine and pyrimidine metabolism were the most highly affected biological pathways in nitrogen-limiting conditions, indicating that these conditions affect energy availability within cells. This stress leads to a shift in cells to the utilization of a less ATP-dependent biological pathway. This information will be beneficial for the development of *Yarrowia* strains for further scientific and industrial applications.

## 1. Introduction

*Yarrowia* is a fungal genus in the family Dipodascaceae. This genus was initially considered monotypic, containing only *Yarrowia lipolytica*, which has been used as a model oleaginous organism for a wide range of studies, including studies of lipid accumulation and degradation, peroxisome biogenesis, dimorphism, and protein secretion pathways [1]. Wild-type *Y. lipolytica* can utilize glucose, fructose, glycerol, and hydrophobic substrates as carbon sources [2,3]. Owing to this property, the yeast species has potential applications in industrial microbiology, particularly for the biosynthesis of lipids [4]. Based on molecular phylogenetics studies, the genus now includes several additional species [5,6,7].

Altering the carbon-to-nitrogen (C:N) ratio in the culture medium could drastically change the metabolomic profile in yeasts, and the precise effects may vary among different strains [8,9,10,11]. Substantial work over the past few years has focused on characterizing and engineering oleaginous yeast [12]. Nonetheless, the metabolic response of *Yarrowia* spp., especially the recently added species, under nitrogen-limiting conditions is not well characterized. The nitrogen-limiting condition is a metabolic condition. Therefore, a complete understanding of its effects cannot be achieved without studying intracellular metabolites [13].

In this study we conducted the time-course sampling of one *Y. lipolytica* strain to probe the suitable time points for multiple strains study. Since the variable changes in this study highly affect the growth of *Y. lipolytica*, it might not be suitable to choose the sampling point based on the growth phase alone. Therefore, we considered the metabolome profile to help understand the dynamic of metabolic changes occuring during the cultivation with several different C:N ratios and determined the crucial sampling time point and C:N ratio for multiple strains study. We further studied the metabolic responses of several *Yarrowia* strains to nitrogen-limiting conditions, including one laboratory strain, *Y. lipolytica* PO1d [14], three former *Candida lipolytica* species from different study groups (currently identified as *Yarrowia* spp.), *C. lipolytica* JCM 2304 [15], *C. lipolytica* JCM 21924 [16], and *C. lipolytica* JCM 8061, and two recently identified species, *Y. deformans* JCM 1694 [17] and *Y. keelungensis* JCM 14894 [18]. This information will be beneficial for the development and manipulation of *Yarrowia* spp. for future applications.

## 2. Results and Discussion

### 2.1. Growth of Y. Lipolytica under Different Carbon-to-Nitrogen Ratios

**Figure 1:**
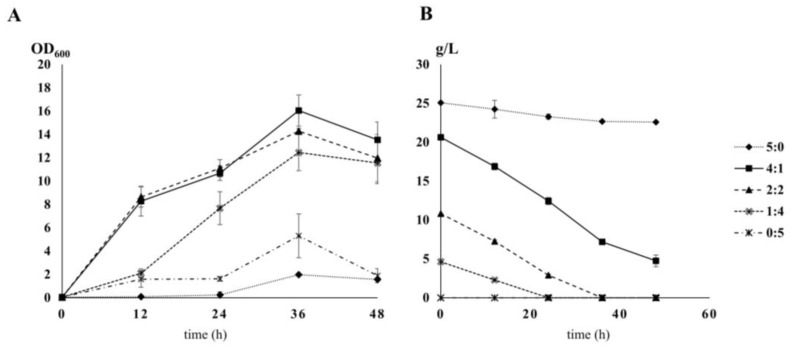
Growth curves of *Y. lipolytica* PO1d and glucose consumption for different carbon-to-nitrogen ratios. *Y. lipolytica* cultures at a starting an OD_600_ of 0.5 for various carbon-to-nitrogen ratios. Error bars represent the standard deviation from three replicates; 5:0 (diamonds with a dashed line), 4:1 (squares with a dark line), 2:2 (triangle), 1:4 (double crosses with a dashed line), 0:5 (asterisks with a dark line). (**A**) Growth curve determined by optical density at 600 nm. (**B**) Glucose (g/L).

To determine the crucial sampling time point and C:N ratio for analyses of the effect of nitrogen-limiting conditions, time course sampling under various C:N compositions was performed using *Y. lipolytica* PO1d. The extracellular glucose concentrations and growth of *Y. lipolytica* cultivated with different C:N ratios are shown in Figure 1. There were three different stable physiological states: (1) Carbon-restricted conditions (0:5), (2) intermediate C:N ratios (4;1, 2:2, and 1:4), and (3) nitrogen-restricted conditions (5:0); all statistical test results can be found in Table S1. Under carbon-restricted conditions, growth increased steadily from 0 h to 12 h, remained constant for 24 h, and cells reached the highest OD_600_ at 36 h. For intermediate C:N ratios, growth increased rapidly from 0 h to 12 h, slowed from 12 h to 24 h, and the highest OD_600_ was reached at 36 h. Under nitrogen-restricted conditions, growth increased slowly from 0 h to 24 h and increased steadily from 24 h until cells reached the highest OD_600_ at 36 h. In this study, all OD_600_ values decreased after 36 h of cultivation. In both carbon- and nitrogen-restricted conditions, the growth profiles suggested that cells took approximately 24 h to adjust to the new condition before entering the exponential growth phase. For intermediate C:N ratios, especially 4:1 and 2:2, three phases of cell growth were observed, 0–12 h, 12–24 h, and 24–36 h. These data suggest that the conditions changed during cultivation, and cells had to adapt for increased growth. Interestingly, this phenomenon was not observed for a C:N ratio of 1:4. These results show that *Y. lipolytica* is very sensitive to changes in the C:N ratio. Surprisingly, *Y. lipolytica* growth did not depend on glucose availability, as evidenced by the continued growth in glucose-depleted conditions (C:N 0:5 and 1:4 at 24 h, 2:2 at 36 h). Yeast in the C:N 5:0 condition could not utilize glucose effectively, even with abundant glucose. Moreover, *Y. lipolytica* entered the death phase, regardless of glucose availability (C:N 4:1, 5:0).

### 2.2. Intracellular Metabolome Analysis of Y. Lipolytica for Different Carbon-to-Nitrogen Ratios

A total of 93 metabolites were annotated (Table S2). The time-course results for all detected metabolites are summarized in Figure S1. Annotated compounds mainly consisted of organic acids, sugars, amino acids, phosphate compounds, and other metabolites. Widely targeted LC-MS/MS data were analyzed by a principal component analysis (PCA) to differentiate among cultivation conditions. The PCA score plot offers a visual image of sample variation from a global view, and, as a non-parametric analysis, the created model is independent of the user; therefore, it is unsupervised [19,20,21].

**Figure 2:**
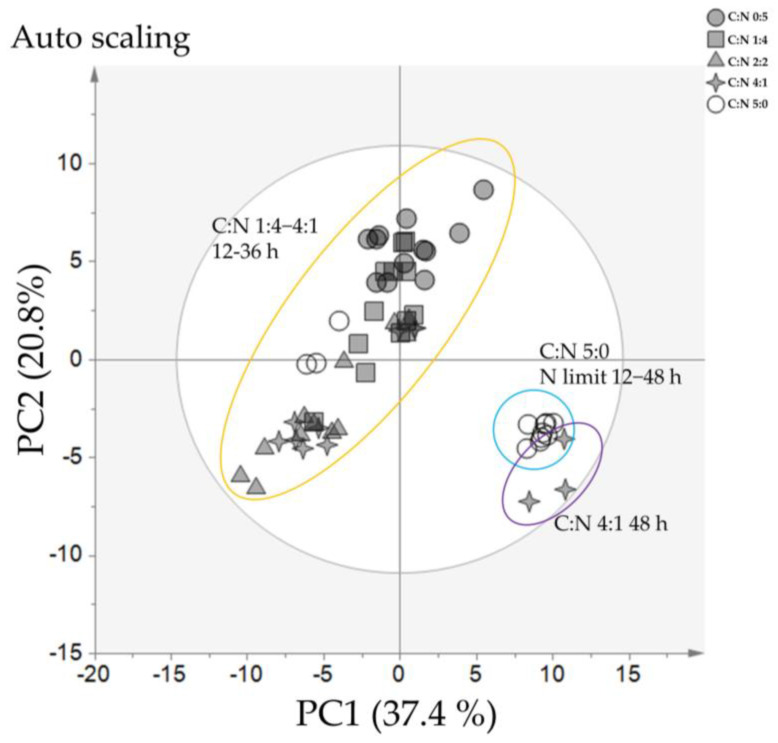
Summary of principal component analysis (PCA) of *Y. lipolytica* cultivated under different carbon-to-nitrogen ratios. The PCA score plot indicates differences in metabolite profiles for different carbon-to-nitrogen ratios based on 93 metabolites. The grey ellipse indicates a 95% confidence border based on Hotelling’s T2. Each point represents one replicate for each C:N ratio; 0:5 (grey circles), 1:4 (grey squares), 2:2 (grey triangles), 4:1 (grey stars), 5:0 (white circles). The yellow ellipse indicates the samples from C:N 1:4−4:1 at 12–36 h, The cyan ellipse indicates the samples from C:N 5:0 at 12–48 h, The purple ellipse indicates the samples from C:N 4:1 at 48 h. The complete numerical PCA loading scores are presented in Table S3.

**Figure 3:**
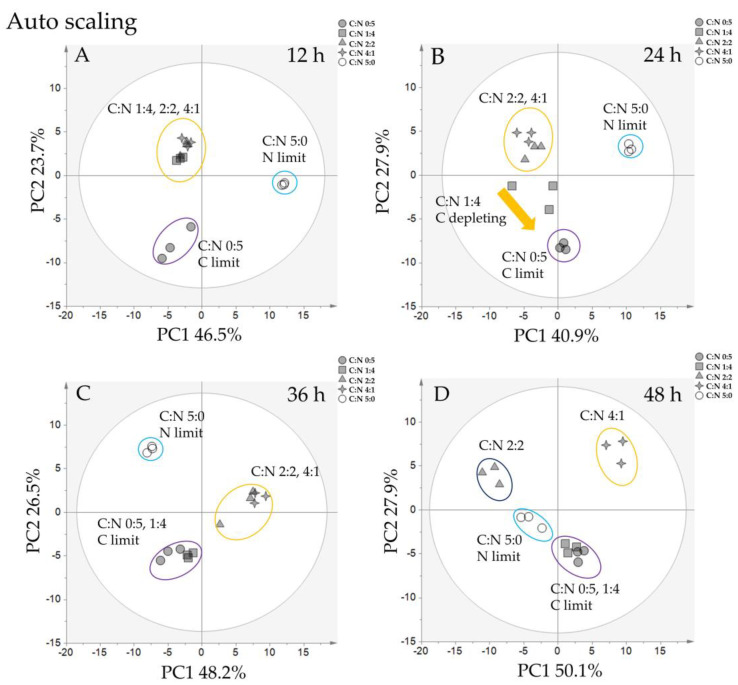
PCA results for *Y. lipolytica* cultivated in different carbon-to-nitrogen ratios. PCA score plot indicating differences in metabolite profiles for different carbon-to-nitrogen ratios based on 93 metabolites. The grey ellipse indicates a 95% confidence border based on Hotelling’s T2. Each point represents one replicate in each condition; 0:5 (grey circle), 1:4 (grey square), 2:2 (grey triangle), 4:1 (grey star), 5:0 (white circle). (Scale: Auto-scale; *n* = 3); (**A**) profile at 12 h, (**B**) profile at 24 h, (**C**) profile at 36 h, and (**D**) at profile at 48 h. The cyan ellipse indicates the nitrogen-limited samples, the purple ellipse indicates the carbon-limited samples, the yellow and blue ellipse indicates samples in which neither carbon nor nitrogen were limited. The full list of PCA loading scores is presented in Table S4.

As shown in Figure 2, when all samples at all time points were evaluated by PCA, two main clusters were separated along PC1. Cluster one included samples with C:N ratios of 0:5, 1:4, 2:2, and 4:1 at 12 h to 36 h of cultivation. Cluster two separated samples into two sub-clusters; sub-cluster one included samples with a C:N ratio of 5:0 at 12 h to 48 h, and sub-cluster two included samples obtained under a C:N ratio of 4:1 at 48 h. These results showed that the most important factor separating the samples was the nitrogen-limiting condition; the C:N ratio of 4:1 at 48 h clustered with the C:N ratio of 5:0, suggesting that at 48 h of cultivation, the nitrogen source with a C:N ratio of 4:1 was depleted. To comprehensively analyze the data, we evaluated each time point separately, as summarized in Figure 3.

As shown in Figure 3A, there were three distinct clusters at 12 h of cultivation, corresponding to the nitrogen-restricted condition, the carbon-restricted condition, and the condition where both carbon and nitrogen were available. PC1 separated the nitrogen-limiting condition and other conditions, while PC2 separated the carbon-limiting condition and other conditions. Most of the metabolites with high PCA scores (top 10 scores on the positive and negative sides of the PC axis) at 12 h were amino acids involved in the aminoacyl-tRNA biosynthesis pathway. Aminoacyl-tRNAs are substrates for translation and determine the interpretation of the genetic code [22,23]. This might indicate that cells in both nitrogen- and carbon-limiting conditions adjust to the new environment at this time point. At 24 h of cultivation (Figure 3B), the clustering results were similar to those at 12 h, except the C:N 1:4 profile was more similar to that under the carbon-restricted condition in relation to glucose consumption (Figure 1B). This probably reflects glucose depletion in the medium, leading to adjustments in cell metabolism to the carbon-restricted condition, similar to 12 h. PC1 at 24 h separated samples in nitrogen-limiting conditions and other conditions, while PC2 separated the carbon-limiting condition from other conditions. However, the metabolites with a high PCA score in PC1 are made up of sugar-phosphate groups from purine and pyrimidine metabolism. These results suggested that purine and pyrimidine metabolism are essential for the response to nitrogen-limiting conditions. At 36 h of cultivation (Figure 3C), the PCA score plot clearly shows that the C:N 1:4 profile merged with the C:N 0:5 profile, indicating that at this time point, the metabolomic profile for the C:N 1:4 condition completely shifted to that of the carbon-restricted condition. The C:N 2:2 and 4:1 profiles clustered together; presumably, the metabolomic profile at this point represents the condition in which carbon and nitrogen are available. Considering the remaining glucose at this time, in the C:N 4:1 condition (Figure 1B), approximately 10 g/L glucose still remains, while glucose in C:N 2:2 is depleted; this might explain why one of the samples in C:N 2:2 started to shift toward the profile of the carbon-restricted condition. Finally, at 48 h of cultivation (Figure 3D), there were four distinct clusters: C:N 4:1, C:N 2:2, C:N 5:0, and C:N 1:4, 0:5 clusters. At this time point, the growth profile (Figure 1A) suggested that cells entered the death phase, and all glucose was depleted except in C:N 5:0 and 4:1. Interestingly, the C:N 2:2 group did not cluster with the carbon-restricted group, suggesting that metabolism during the death phase is different, even in the carbon- or nitrogen-restricted condition. Metabolites with a high PCA score at 36 h and 48 h were similar to those at 24 h, where PC1 consisted mostly of sugar-phosphate compounds, and PC2 consisted of metabolites from amino acid and central carbon metabolism. Purine and pyrimidine are involved in the synthesis of thiamine, riboflavin, folic acid pteridines, and histidine or are constituents of cytokinins, purine alkaloids, and other unusual nitrogen compounds related to nitrogen accumulation or nitrogen excretion [24,25]. As purine and pyrimidine are nitrogen sources stored in cells, the lack of nitrogen supply in the medium might directly or indirectly affect purine and pyrimidine biosynthesis in yeast. Moreover, purine and pyrimidine and their derivatives are linked to the synthesis, transfer, and utilization of stored energy in energy metabolism [26].

**Figure 4:**
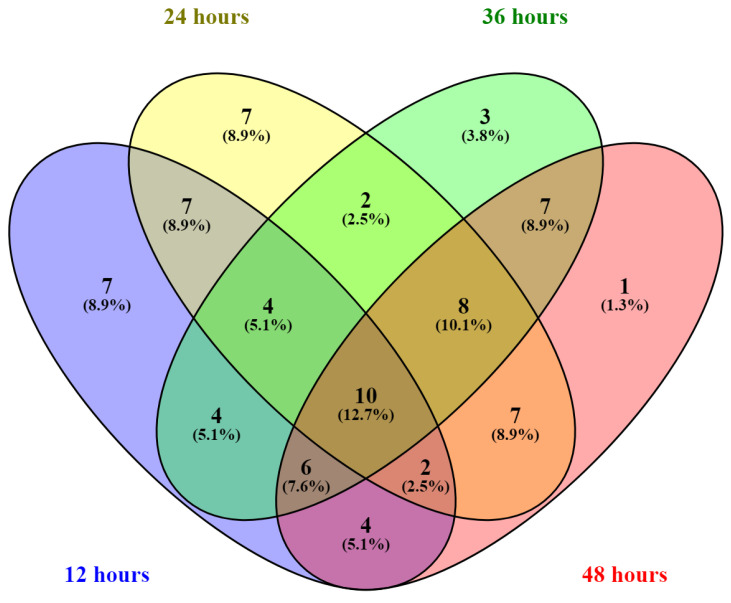
Venn diagram of selected metabolites based on principal component loading scores. The metabolites with high scores from PCA 1 and 2 at each cultivation time were included in the analysis. A list of metabolites in each subset is provided in Table S6.

The principal component loading scores on PC1 and PC2 at each time point was converted to an absolute value to rank their contribution to the separation of the data on the PC scatter plot. Metabolites with a PC score in the upper 75th percentile for the same cultivation time and principal component were selected for Venn diagram construction, as shown in Figure 4. We found ten core metabolites at all cultivation time points, i.e., ADP, arginine, F6P, lysine, trehalose, IMP, BPG, FAD, PEP, and NADP. These metabolites with a high PCA score throughout the cultivation period have a wide range of fluctuation with respect to C:N ratios in every stage of growth. Trehalose is a non-reducing disaccharide found ubiquitously in fungi and is widely found in bacteria and animals [27]. Trehalose was initially thought of as a reserve carbohydrate in yeast. However, its role as a stress protectant in yeast has been recognized [28]. The primary role of trehalose is to protect the cytosol against adverse conditions, such as desiccation, frost, and heat. In the budding yeast *Saccharomyces cerevisiae*, trehalose levels are low during rapid growth periods and increase during periods of slow or no growth [29]. These results agree with our results for *Y. lipolytica* (Figure S2), except in the condition where carbon or nitrogen was restricted. As discussed above, purine and pyrimidine metabolism were affected. Notably, IMP and ADP were among the metabolites with high PCA scores at every time point. It is possible that these metabolites were at the center of the biological reaction in purine metabolism. Therefore, the effect of changes in conditions could be observed in these metabolites. Consequently, FAD, which requires GTP from the purine biosynthesis pathway, was also affected. For arginine, carbamoyl phosphate, an upstream metabolite for arginine biosynthesis, was directly involved in pyrimidine metabolism and nitrogen metabolism. The high PCA score for arginine might reflect the effect of the upstream pathway. PEP, F6P, and BPG are essential intermediates in the TCA cycle, suggesting that glycolysis/gluconeogenesis or related metabolites in the pathway were utilized differently under different C:N ratios; consequently, lysine, which is linked to central metabolism, was also affected. Finally, NADP is a cofactor used in anabolic reactions, such as the Calvin cycle and lipid and nucleic acid syntheses, which require NADPH as a reducing agent. As the carbon and nitrogen sources were interrupted, the NADP level changed drastically. The time course of relative intensities is shown in Figure S2, all *t*-test *p*-value could be found in Table S5. The exact mechanisms of how the nitrogen-limiting condition affects the glucose uptake in *Y. lipolytica* was unclear but we hypothesized that the lower energy molecule might cause the ATP-dependent pathway in *Y. lipolytica* related to carbon metabolisms to slow down.

### 2.3. Intracellular Metabolome Analysis of Different Yarrowia spp. in Normal and Nitrogen-Limiting Conditions

The time-course analysis of the *Y. lipolytica* PO1d metabolomics profile provides information regarding metabolic dynamics with respect to different C:N ratios and time to study the responses of *Yarrowia* strains to nitrogen-limiting conditions. Based on growth, glucose consumption, and metabolomic profile results, we concluded that the appropriate time point for sample collection for this purpose was 36 h, as the metabolomic profile of C:N 4:1 was superimposed with that for C:N 5:0 at 48 h (Figure S3), suggesting that the nitrogen source at 48 h for C:N 4:1 was depleted. In addition, the growth of C:N 5:0 at 24 h was limited. To study the response to nitrogen-limiting conditions, a standard C:N ratio of 4:1 was selected for comparison against the nitrogen-limiting condition of 5:0 as the C:N 5:0 gave the distinct metabolome profile throughout the cultivation. The growth profiles of different strains confirmed that *Yarrowia* spp. have similar standard growth profiles (Figure S4). In this study, we investigated the responses of six *Yarrowia* strains, *Y. lipolytica* PO1d, *Y. deformans* JCM 1694, *Y. keelungensis* JCM 14894, *C. lipolytica* JCM 2304, *C. lipolytica* JCM 21924, and *C. lipolytica* JCM 8061. The complete numerical results of all detected normalized peak areas of six *Yarrowia* spp. in normal and nitrogen-limiting conditions are presented in Table S7.

**Figure 5:**
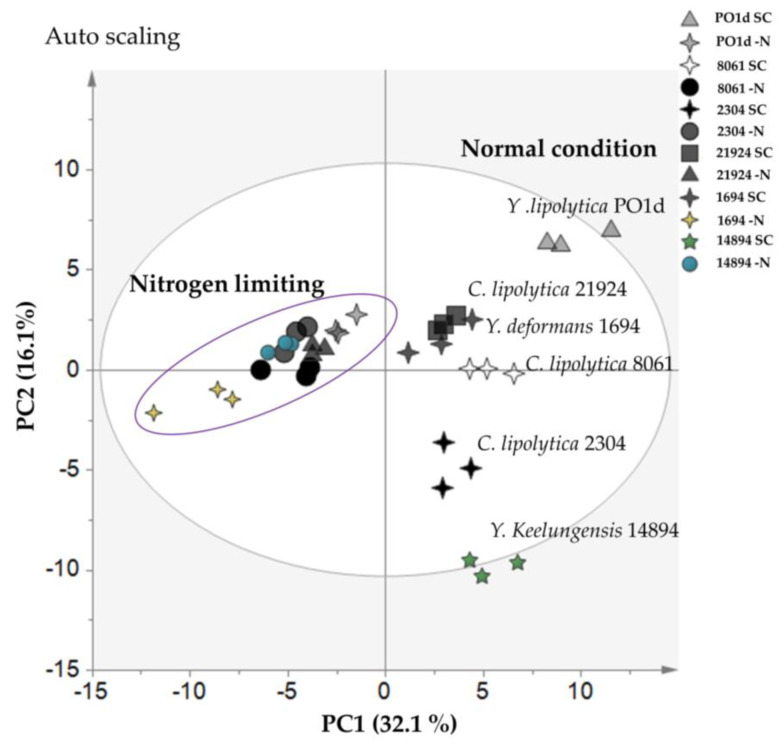
PCA results for six *Yarrowia* spp. cultivated in normal and nitrogen-limiting conditions. PCA score plot indicating differences in metabolite profiles for two carbon-to-nitrogen ratios based on 100 metabolites. The grey ellipse indicates a 95% confidence border based on Hotelling’s T2. The purple ellipse indicates the nitrogen-limited samples. Each point represents one replicate; *Y. lipolytica* PO1d in normal conditions (light grey triangles), *Y. lipolytica* PO1d in nitrogen-limiting conditions (light grey four-point stars), *C. lipolytica* JCM 8061 in normal conditions (white four-point stars), *C. lipolytica* JCM 8061 in nitrogen-limiting conditions (black circles), *C. lipolytica* JCM 2304 in normal conditions (black four-point stars), *C. lipolytica* JCM 2304 in nitrogen-limiting conditions (dark grey circles), *Y. lipolytica* JCM 21924 in normal conditions (dark grey squares), *Y. lipolytica* JCM 21924 in nitrogen-limiting conditions (dark grey triangles), *Y. deformans* JCM 1694 in normal conditions (dark grey four-point stars), *Y. deformans* JCM 1694 in nitrogen-limiting conditions (yellow four-point star), *Y. keelungensis* JCM 14894 in normal conditions (green five-point stars), *Y. keelungensis* JCM 14894 in nitrogen-limiting conditions (blue circles). Right, top 25% of metabolites based on PCA scores from each principal component. The full list of PCA loading scores is presented in Table S8.

As shown in Figure 5, PC1 separated the yeast cultivated under normal and nitrogen-limiting conditions, while PC2 separated each strain of *Yarrowia*. While all of the yeast in this study belonged to the genus *Yarrowia*, the results suggested that each *Yarrowia* strain has a unique metabolomic profile, including variation at the subspecies level. The high PC score for PC1 agrees with the initial analysis of *Y. lipolytica* PO1d, in which most of the metabolites with high PC scores were from purine and pyrimidine metabolism, suggesting that *Yarrowia* spp. respond to nitrogen-limiting conditions in a similar way.

**Figure 6:**
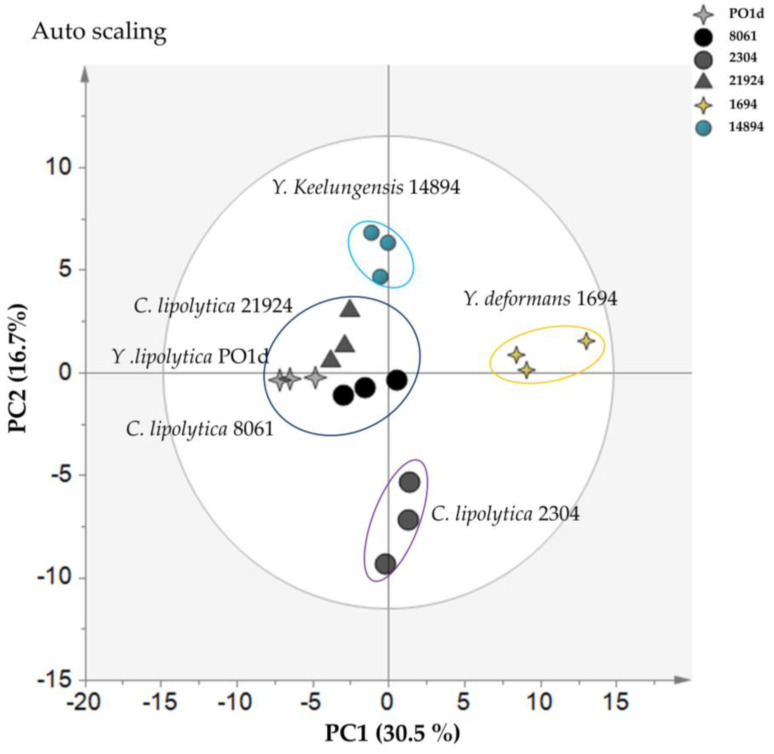
PCA results for six *Yarrowia* spp. cultivated in normal and nitrogen-limiting conditions; (left) PCA score plot indicates differences in metabolite profiles among strains in the nitrogen-limiting condition based on 100 metabolites. The grey ellipse indicates a 95% confidence border based on Hotelling’s T2. The cyan ellipse indicates samples from *Y. keelungensis* JCM 14894, the yellow ellipse indicates samples from *Y*. *deformans* JCM 1694, the purple ellipse indicates samples from *C. lipolytica* JCM 2304, the blue ellipse indicates samples from *C. lipolytica* JCM 21924, 8061 and *Y. lipolytica* PO1d. Each point represents one replicate; *Y. lipolytica* PO1d (light grey four-point stars), *C. lipolytica* JCM 8061 (black circles), *C. lipolytica* JCM 2304 (dark grey circles), *C. lipolytica* JCM 21924 (dark grey triangles), *Y. deformans* JCM 1694 (yellow four-point stars), and *Y. keelungensis* JCM 14894 (blue circles); (right) top 25% of metabolites based on PCA scores from each principal component. The full list of PCA loading scores is presented in Table S8.

We further analyzed differences among *Yarrowia* strains under nitrogen-limiting conditions. As shown in Figure 6, PC1 separated *Y. deformans* JCM 1694 and the remaining taxa, while PC2 separated the following three groups: (1) *Y. keelungensis*, JCM 14894 (2) *Y. lipolytica* PO1d, *C. lipolytica* JCM 21924, and *C. lipolytica* JCM 8061, and (3) *C. lipolytica* JCM 2304. The high PC1 score suggested that *Y. deformans* JCM 1694 has a different response to nitrogen-limiting conditions than those of other *Yarrowia* spp. Interestingly, PC2 separated not only the different *Yarrowia* species but also the subspecies *C. lipolytica* JCM 2304; this result shows that *Yarrowia* is a highly diverse genus even at the subspecies level. The results for all detected metabolites are summarized in Figure S5.

Surprisingly, the metabolites from PC2 include MEP and MEcPP; (Table S8) these metabolites are in the MEP pathway, which has not been reported in this yeast genus. However, Soliman et al. [30] detected a MEP-like pathway in fungi. We hypothesized that the nitrogen-limiting condition reduces purine and pyrimidine activity in yeast, which affects the energy availability in the cell. This stress leads to a cellular adjustment to the less ATP-dependent pathway, i.e., a switch from the MVA pathway to the MEP pathway, which requires less ATP. However, further biological investigations are required.

**Figure 7:**
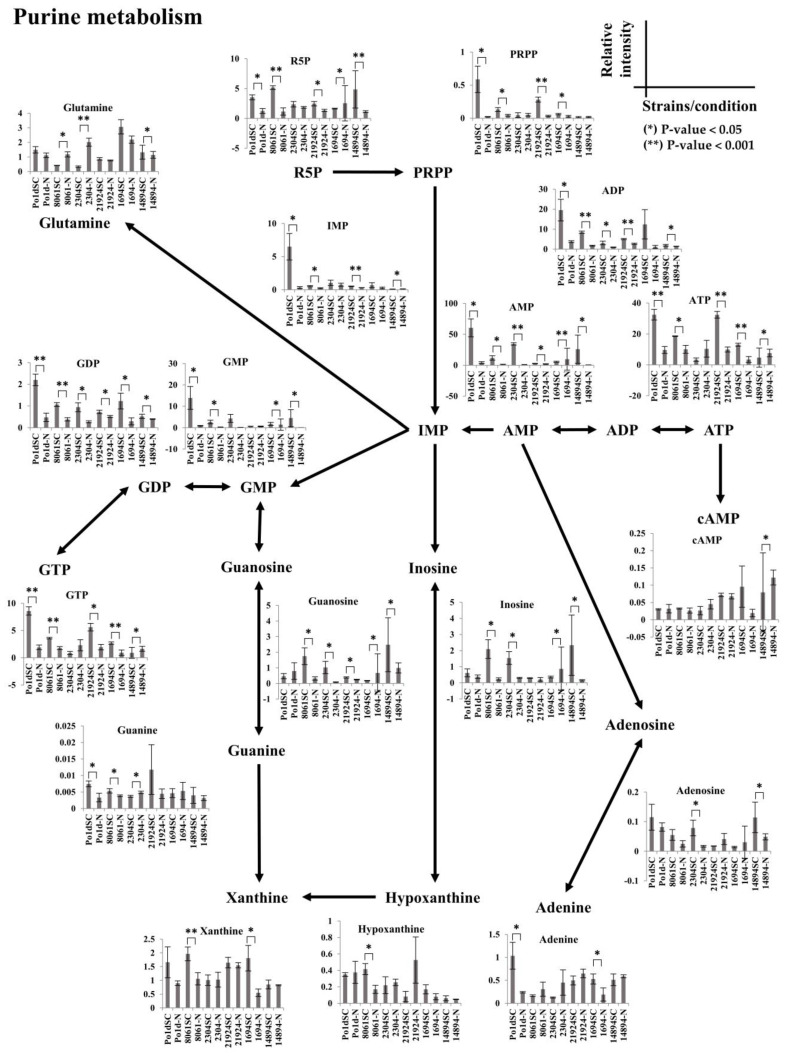
Normalized peak areas for all detected metabolites in purine metabolism. The pathway skeleton was obtained from the KEGG pathway database [31]. SC indicates C:N 4:1, -N indicates C:N 5:0. Error bars indicates standard deviation, * indicates *p*-value < 0.05, ** indicates *p*-value < 0.001.

**Figure 8:**
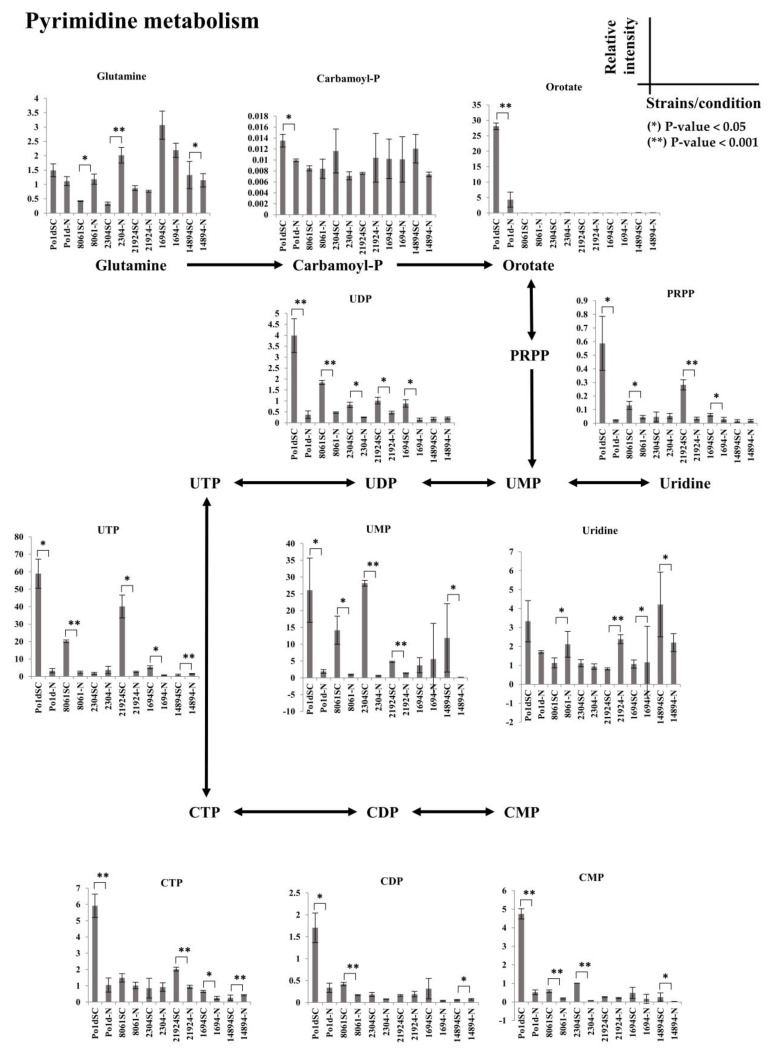
Normalized peak areas for all detected metabolites in pyrimidine metabolism. The pathway skeleton was obtained from the KEGG pathway database. SC indicates C:N 4:1, -N indicates C:N 5:0. Error bars indicates standard deviation, * indicates *p*-value < 0.05, ** indicates *p*-value < 0.001.

Peak areas for all detected metabolites of purine and pyrimidine metabolism are summarized in Figure 7 and Figure 8, respectively, all *t*-test *p*-value could be found in Table S9. These results clearly demonstrate that *Yarrowia* strains respond to the nitrogen-limiting condition in a similar way; notably, R5P, ADP, ATP, GDP, and GTP in purine metabolism were similar among strains. On the other hand, each species exhibited distinct metabolomic profiles for other metabolites in purine metabolism and all other detectable metabolites in pyrimidine metabolism. The laboratory *Y. lipolytica* PO1d strain had a distinct profile in which the orotate, PRPP, IMP, CTP, CDP, and CMP levels were exceptionally high compared to those in other strains. In general, the levels of metabolites in purine and pyrimidine metabolism were lower under nitrogen-limiting conditions than under normal conditions; however, *C. lipolytica* JCM 8061 had an interesting pattern in which metabolite profiles in the nitrogen-limiting condition were reversed compared to those of other strains, including glutamine, adenine, and uridine. *C. lipolytica* JCM 2304 had a unique profile in which many metabolites in purine and pyrimidine metabolism were not affected by nitrogen-limiting conditions, including R5P, guanine, cAMP, xanthine, hypoxanthine, UTP, uridine, and PRPP. *C. lipolytica* JCM 21924 metabolites under nitrogen-limiting conditions, including adenine and uridine, showed the opposite patterns compared to those of other strains; however, unlike JCM 8061, glutamine was not affected. *Y. deformans* JCM 1694 had the highest glutamine content, and levels of all metabolites in purine and pyrimidine metabolism were lower in the nitrogen-limiting condition. *Y. keelungensis* JCM 14894 was the only strain in which metabolites from purine metabolism were mostly unaffected; however, metabolites of pyrimidine metabolism were quite similar to those of other strains. The different profiles of *Yarrowia* spp. observed in this study can be utilized for metabolic manipulations for special applications in the future. Based on these results, to engineer microorganisms for a particular application, it is necessary to consider the precise metabolic traits of the strain for optimization. We demonstrated that metabolomics is a powerful tool for this purpose.

## 3. Materials and Methods

### 3.1. Yeast Strains, Cultivation, and Sample Collection

The *Y. lipolytica* laboratory strain PO1d was obtained from the National University of Singapore, *C. lipolytica* JCM 2304, *C. lipolytica* JCM 21924, *C. lipolytica* JCM 8061, *Y. deformans* JCM 1694, and *Y. keelungensis* JCM 14894 were obtained from the Japan Collection of Microorganisms (JCM). Yarrowia strains were pre-cultivated overnight in synthetic complete (SC) medium, all components were prepared according to the manufacturer’s instruction (20 g/L glucose, 1.7 g/L, yeast nitrogen-based without amino acids and ammonium sulfate purchased from BD Difco product number 233520, 5 g/L ammonium sulfate, drop-out medium supplements purchased from Sigma-Aldrich product number Y1501 consists of all standard amino acids at 76 mg/L except for leucine, which is present at 380 mg/L, adenine (18 mg/L), inositol (76 mg/L), p-aminobenzoic acid (8 mg/L) in 100-mL shake flasks at 30 °C and 200 rpm. The pre-cultured cells were inoculated into 20 mL of SC medium with different C:N ratios (e.g., C:N ratio 4:1 consists of 20 g/L glucose, 5 g/L ammonium sulfate, all standard amino acids at 76 mg/L except for leucine, which is present at 380 mg/L, 18 mg/L adenine, 76 mg/L inositol, 8 mg/L p-aminobenzoic acid, 1.7 g/L, yeast nitrogen-based without amino acids and ammonium sulfate. C:N 5:0 consists of 25 g/L glucose, all standard amino acids at 76 mg/L except for leucine, which is present at 380 mg/L, 18 mg/L adenine, 76 mg/L inositol, 8 mg/L p-aminobenzoic acid, 1.7 g/L, yeast nitrogen-based without amino acids and ammonium sulfate) to obtain an initial optical density at 600 nm of 0.5 in 100-mL shake flasks at 30 °C and 200 rpm. For sample collection, cells were collected at an OD_600_ of 5.0 at 12, 24, 36, and 48 h by fast filtration using a 0.45-µm pore size, 47-mm diameter nylon membrane (Millipore, Billerica, MA, USA). The cells were immediately immersed in liquid nitrogen for quenching and stored at −80 °C until extraction.

### 3.2. Fast Filtration and Extraction of Intracellular Metabolites

Before LC-MS/MS, pre-analysis processes, such as quenching and storing the samples at −80 °C, were performed. Subsequently, data acquisition and analysis were performed to determine the relative concentration of each metabolite. The acquired signal intensities of metabolites were compared among samples after the normalization process. Here, the samples were normalized using champorsulfonic acid. For extraction, 1.8 mL of extraction solvent (methanol/water/chloroform = 5:2:2 v/v/v%, with 20 µg/L camphorsulfonic acid as an internal standard) was added to each 2-mL sampling tube with the filtered sample, followed by incubation at −30 °C for 1 h. After incubation, 700 µL of the solution was transferred to a new tube containing 350 µL of water. The mixture was homogenized by vortexing and centrifuged at 9390× *g*. for 10 min at 4 °C to separate polar and non-polar phases. Then, 700 µL of the upper polar phase was transferred to a new tube via syringe filtration (0.2-µm PTFE hydrophilic membrane; Millipore). The sample was centrifugally concentrated for 2 h and freeze-dried overnight. After reconstitution in 50 µL of ultrapure water, the sample was centrifuged at 16,000× *g* for 3 min at 4 °C and transferred to a glass vial for the LC/MS analysis.

### 3.3. Analysis of Intracellular Metabolites by LC-MS/MS

The samples were analyzed based on the method adopted from Dempo et al. [32]. Briefly, ion-pair reversed phase LC/MS/MS was carried out using a Nexera UHPLC system (Shimadzu, Kyoto, Japan) coupled with LCMS 8030 Plus (Shimadzu) for the time course analysis and coupled with LCMS 8050 (Shimadzu) for analyses of multiple strains. The column was a CERI (Chemicals Evaluation and Research Institute, Tokyo, Japan) L-column 2 metal-free ODS (150 mm 2.1 mm, particle size 3 µm). Mobile phase (A) was 10 mM tributylamine and 15 mM acetate in ultra-pure water, and mobile phase (B) was pure methanol. The flow rate was set at 0.2 mL/min, and the column oven temperature was set at 45 °C. The concentration of mobile phase (B) was programmed to increase from 0% to 15%, 50%, and 100% from 1.0 to 1.5 min, 3.0 to 8.0 min, and 8.0 to 10.0 min, respectively, held until 11.5 min, then decreased to 0% from 11.5 min and held at 0% for 20 min. The analysis mode was set to negative ion detection mode. The injection volume was 3 µL, desolvation line temperature was set at 250 °C, probe position was +1.5 mm, heat block temperature was set at 400 °C, nebulizer gas flow was set at 2 L/min, and drying gas flow was set at 15 L/min.

### 3.4. Multivariate Analysis

Data analysis was carried out by converting the raw data from Shimadzu file format (.lcd) to an analysis base file (.abf) format using an open source file converter (Reifycs Inc., Tokyo, Japan). After data conversion, MRMPROBS 2.19 [33] was used for automatic peak selection and peak area integration. The detected peaks were manually confirmed using Lab Solution (Shimadzu Co.). A principal component analysis (PCA) was carried out using SIMCA-P+ version 13 (Umetrics, Umeå, Sweden). The metabolome data were normalized using an internal standard, mean-centered, and scaled to unit variance. A *t*-test to determine statistically significant differences was performed using MS Excel.

### 3.5. Glucose Measurement

The remaining glucose in the culture broth was determined using the GL Science GL-7400 high-performance liquid chromatography system coupled with a column oven GL-7432 and refractive index detector GL-7454 (HPLC-RI). The column was Shim-pack SPR-Pb (250 mm length and ID 7.8 mm (Shimadzu). The supernatant of the culture broth was centrifuged at 10,000× *g* for 5 min at 4 °C; 300 µL of the sample was filtered in a Whatman syringeless LC vial (Merck, Kenilworth, NJ, USA). The sample injection volume was 10 µL; the oven temperature was set to 80 °C. Ultrapure water was used as the mobile phase with a 0.6 mL/min flow rate and 189 psi of back pressure.

## 4. Conclusions

A metabolomics analysis revealed that *Yarrowia* is a very diverse genus, although the response to the nitrogen-limiting condition is similar among strains. However, despite the similarity, each strain had a unique metabolome profile. Nitrogen-limiting conditions cause lower purine and pyrimidine activity in *Yarrowia* spp., which affected the availability of energy molecules in the cell. This stress might lead to the adjustment to a less ATP-dependent pathway. This information will be beneficial for the development of *Yarrowia* strains for further scientific and industrial applications.

## Data Availability

The data presented in this study are available in the supplementary data and Supplementary Tables S1–S9.

## Supplementary Materials

The following are available online at https://www.mdpi.com/2218-1989/11/1/16/s1, Figure S1: Heat map describes the time-course change of the normalized peak areas metabolome profile of 93 metabolites, Figure S2: Line chart of core metabolites extracted from top 25 percent of PC1 and PC2 from 12 to 48 h of cultivation error bars indicates standard deviation, Figure S3: All-time point score plot (A) all sample from 12–36 h of cultivation (B) all sample from 12–48 h of cultivation, Figure S4: Growth profile comparing *Y. lipolytica* spp. error bars indicates standard deviation, Figure S5: Heat map describes the average normalized peak areas of the metabolome profile of 100 metabolites from six *Yarrowia* spp. cultivated in two conditions. Table S1: *p*-value from *t*-test of growth and glucose consumption profile; yellow highlight indicates *p*-value < 0.05, Table S2: complete numerical results of normalized peak area in the time-course sampling of *Y. lipolytica* PO1d in different C:N ratio, Table S3: complete numerical results of PCA loading score from all samples of *Y. lipolytica* PO1d in different C:N ratio. Table S4: complete numerical results of PCA loading score from each time point of *Y. lipolytica* PO1d in different C:N ratio. Table S5: *p*-value from *t*-test of core metabolites line chart in Figure S2; yellow highlight indicates *p*-value < 0.05, Table S6: complete results of Venn diagram analysis from Figure 4, Table S7: complete numerical results of all detected normalized peak area of six *Yarrowia* spp. in normal and nitrogen-limiting conditions, Table S8: complete numerical results of PCA loading score of six *Yarrowia* spp. in normal and nitrogen-limiting conditions. Table S9: *p*-value from *t*-test of Figure 7 and Figure 8; yellow highlight indicates *p*-value < 0.05.
