# Supplementary material for: Metabolomic Analysis of Response to Nitrogen-Limiting Conditions in Yarrowia spp
Source: Metabolites. 2020 Dec 29;11(1):16. doi: 10.3390/metabo11010016 (PMC7823547; doi:10.3390/metabo11010016)
Supplement: Supplementary file 1 [file metabolites-11-00016-s001.zip › Supplementary_Material.docx]

**Supplementary Material**


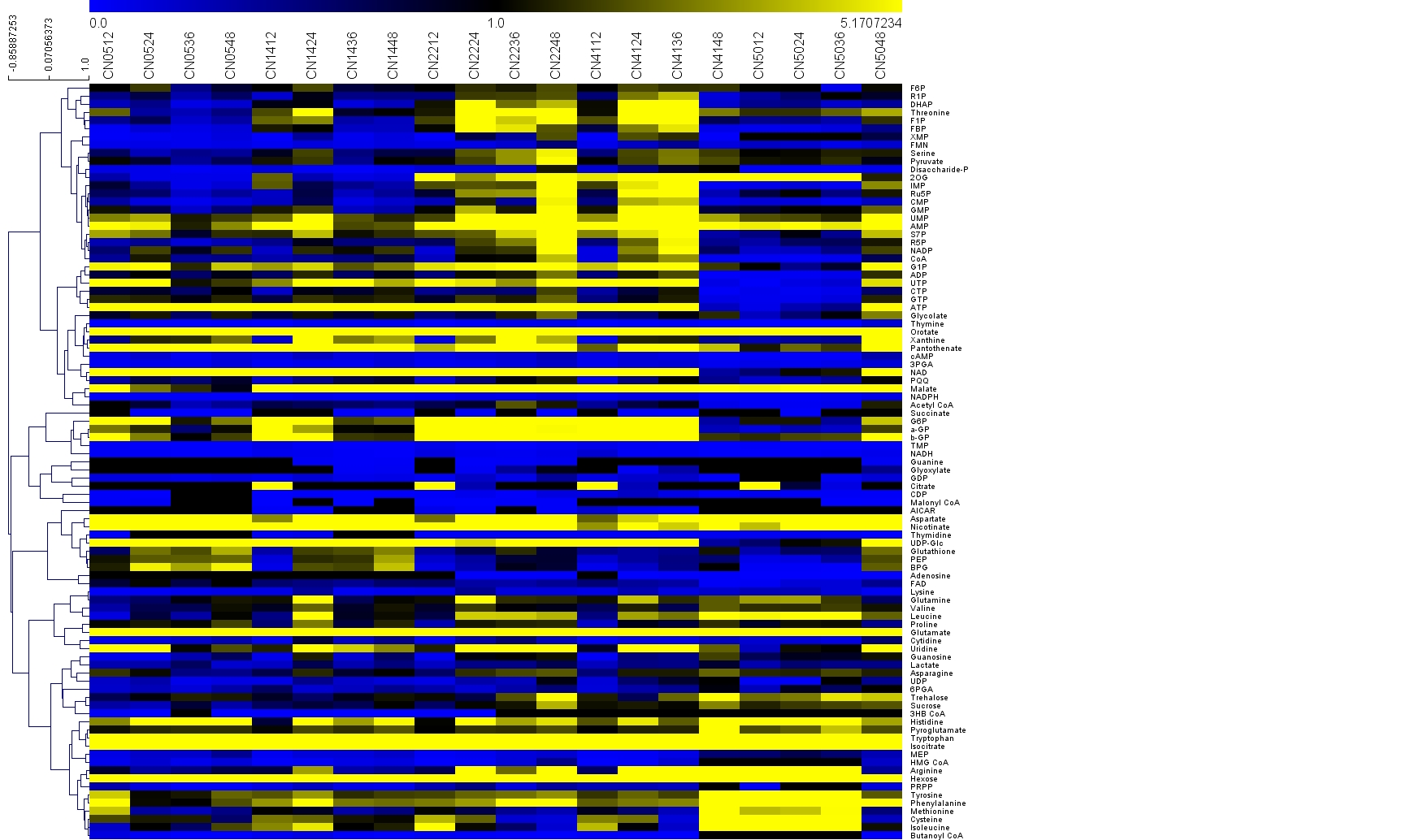


**Figure S1**. Heat map describes the time-course change of the normalized peak areas metabolome profile of 93 metabolites from *Y. lipolytica* PO1d was clustered hierarchically by a complete linkage method using the MeV software ver. 4.90.


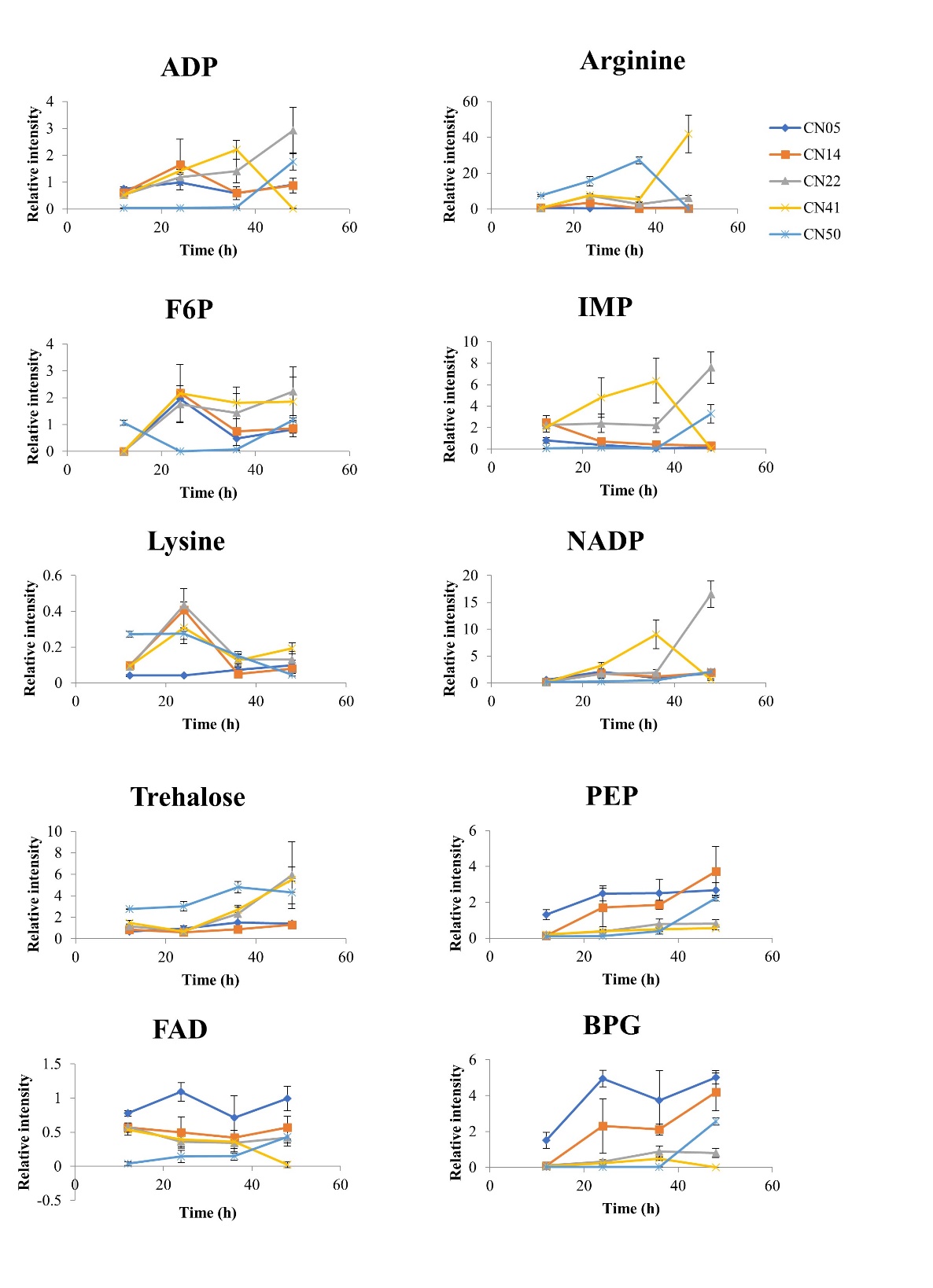


**Figure S2.** Line chart of core metabolites. extracted from top 25 percent of PC1 and PC2 from 12 to 48 hours of cultivation Error-bar represent standard deviation from three replicates; 0:5 (diamond with blue line), 1:4 (square with orange line), 2:2 (triangle with grey line), 4:1 (cross with), 5:0 (asterisk with light blue line). T-test p-values table for all tested condition could be found in Table S5.


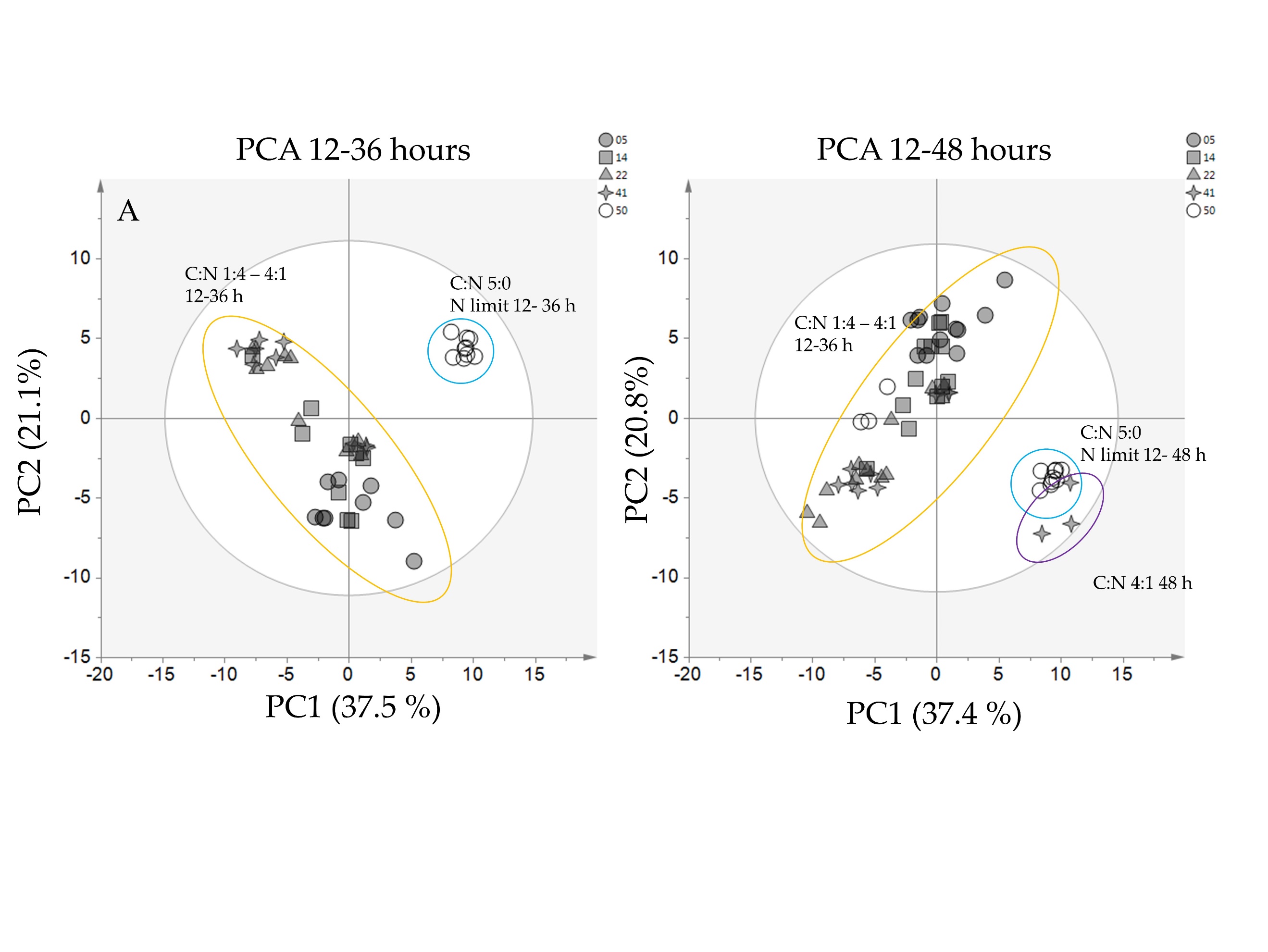


**Figure S3**. All-time point score plot (A) all samples from 12 – 36 hours of cultivation (B) all samples from 12 – 48 hours of cultivation. The results show that the C:N 4:1 group with the nitrogen condition at 48 hours, therefore the 36 hour time point is the last suitable time point for nitrogen limiting comparisons.

Time (h)

**Figure S4**. Growth profile comparing *Y. lipolytica* spp*.* Error-bar represents the standard deviation from three replicates; *Y. deformans* JCM 1694 (square with dot line), *Y. keelungensis* JCM 14894 (triangle with dark line), *C. lipolytica* JCM 21924 (diamond with dash line).


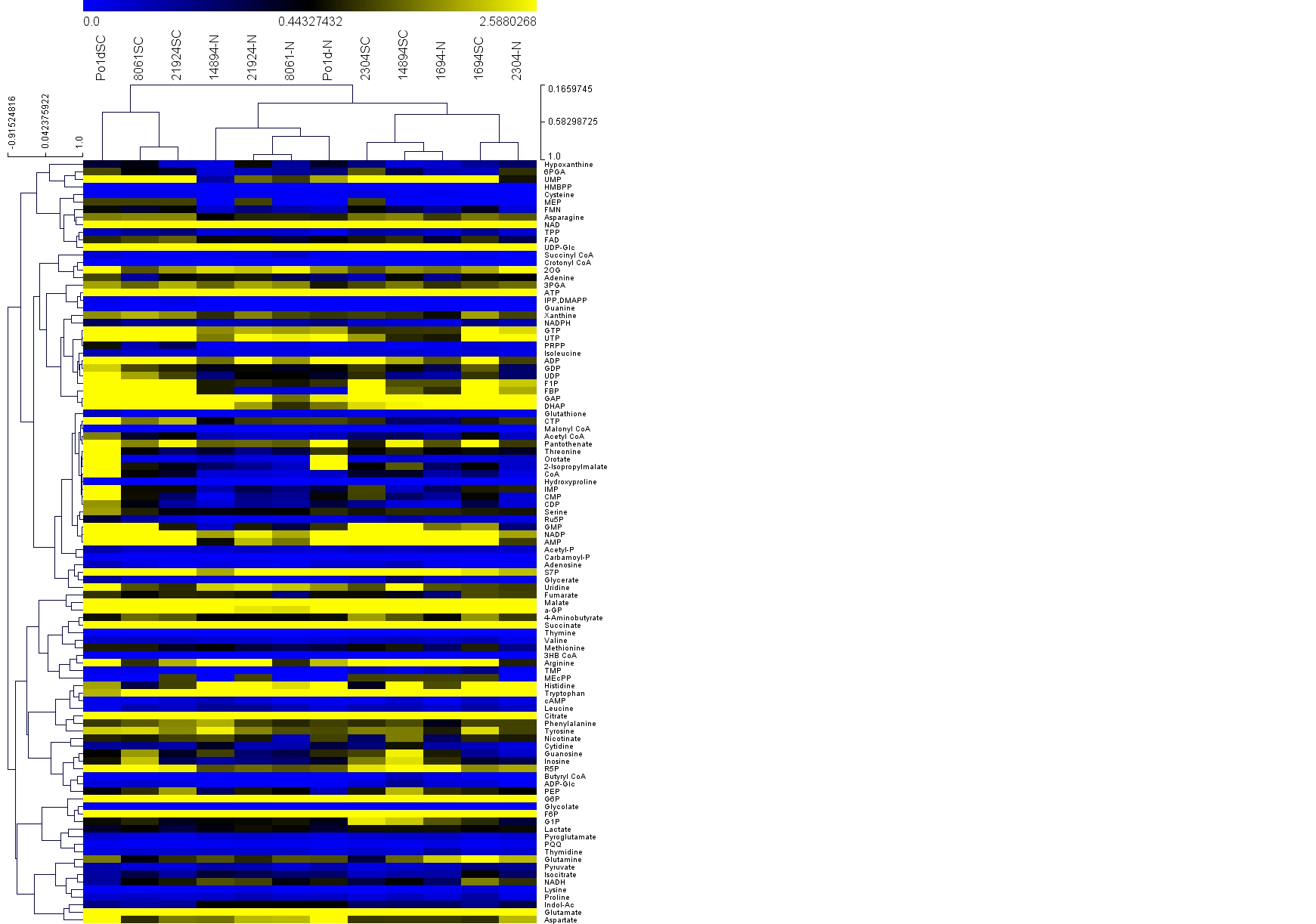


**Figure S5**. Heat map describes the average normalized peak areas metabolome profile of 101 metabolites from 6 *Yarrowia* spp*.* cultivated in two conditions sample was clustered hierarchically by a complete linkage method using the MeV software ver. 4.90. SC indicates synthetic complete medium, -N indicates the removal of ammonium sulfate.
