# Supplementary figures and images for: Metabolomic Analysis of Response to Nitrogen-Limiting Conditions in Yarrowia spp
Source: Metabolites. 2020 Dec 29;11(1):16. doi: 10.3390/metabo11010016 (PMC7823547; doi:10.3390/metabo11010016)

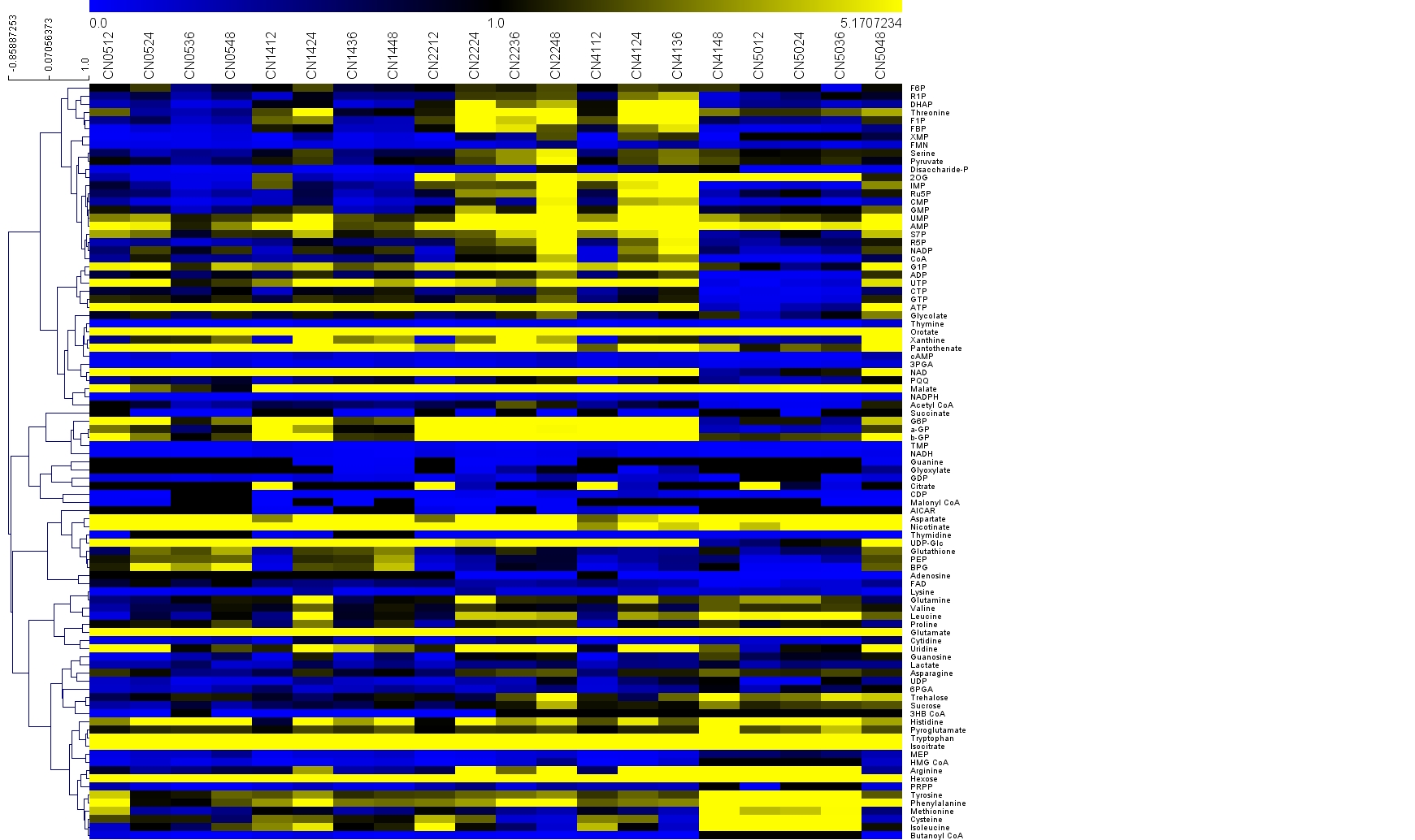

Supplement: Supplementary file 1 [file metabolites-11-00016-s001.zip › Figure S1.jpg]

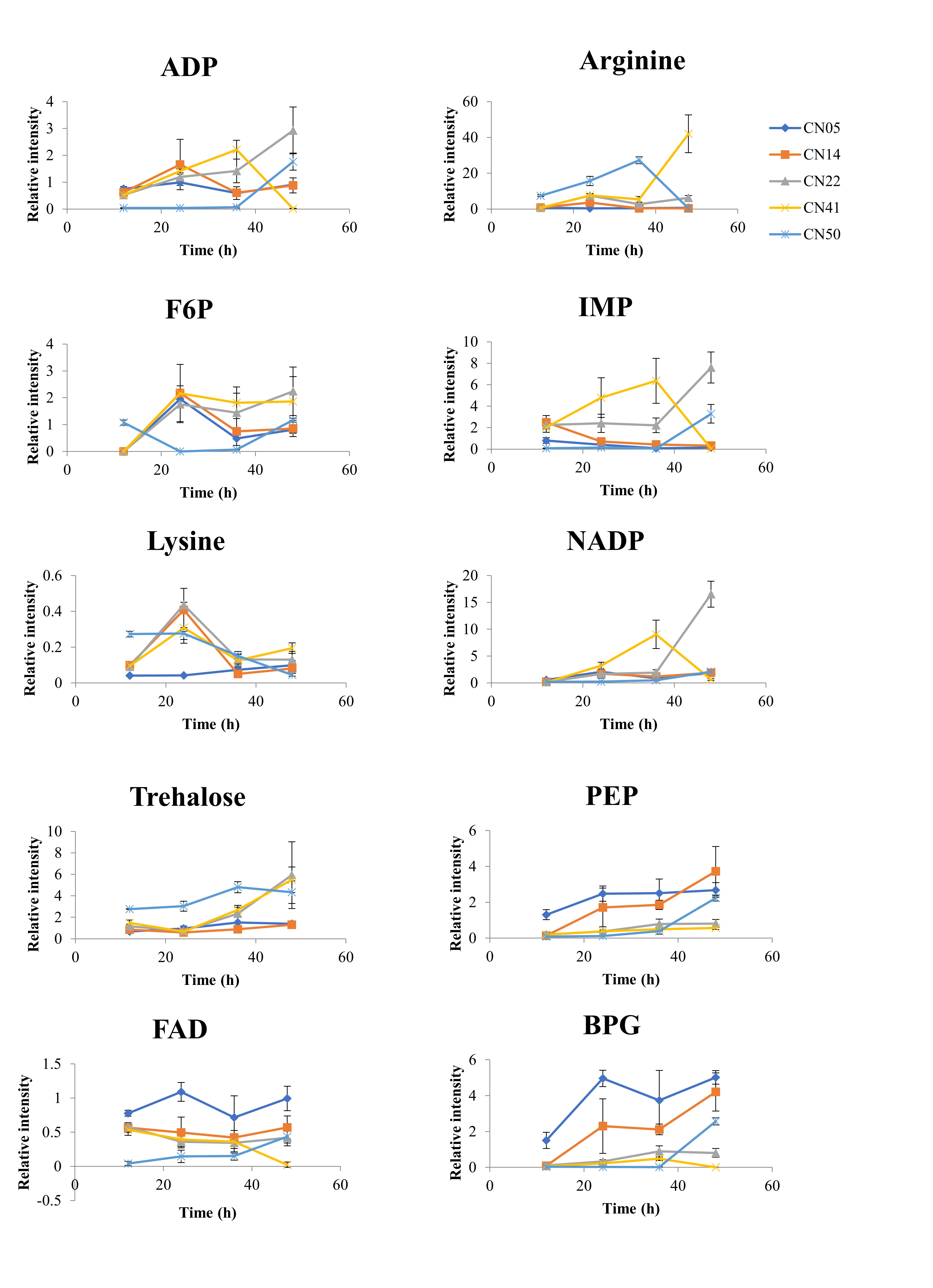

Supplement: Supplementary file 1 [file metabolites-11-00016-s001.zip › Figure S2.JPG]

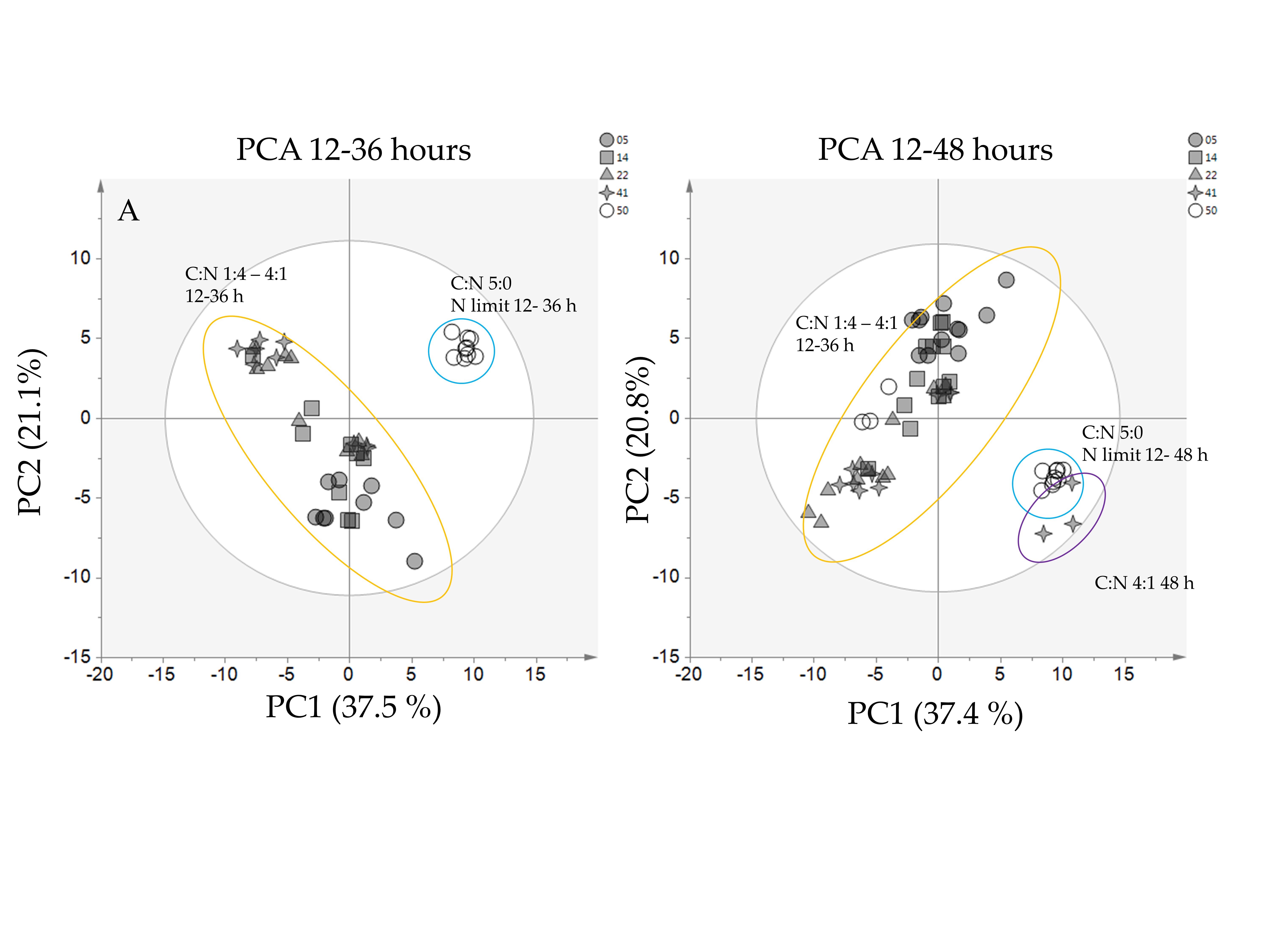

Supplement: Supplementary file 1 [file metabolites-11-00016-s001.zip › Figure S3.jpg]

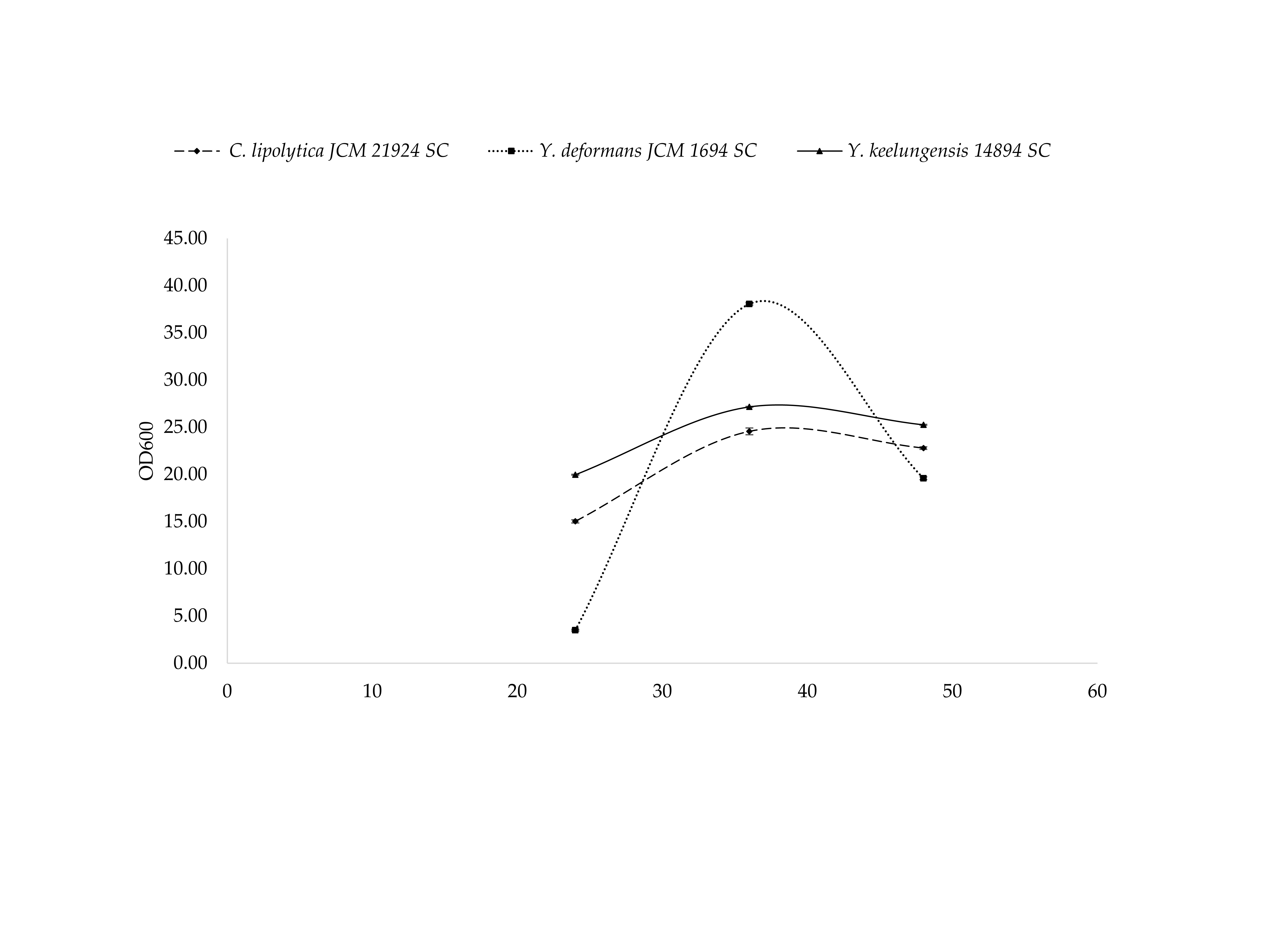

Supplement: Supplementary file 1 [file metabolites-11-00016-s001.zip › Figure S4.jpg]

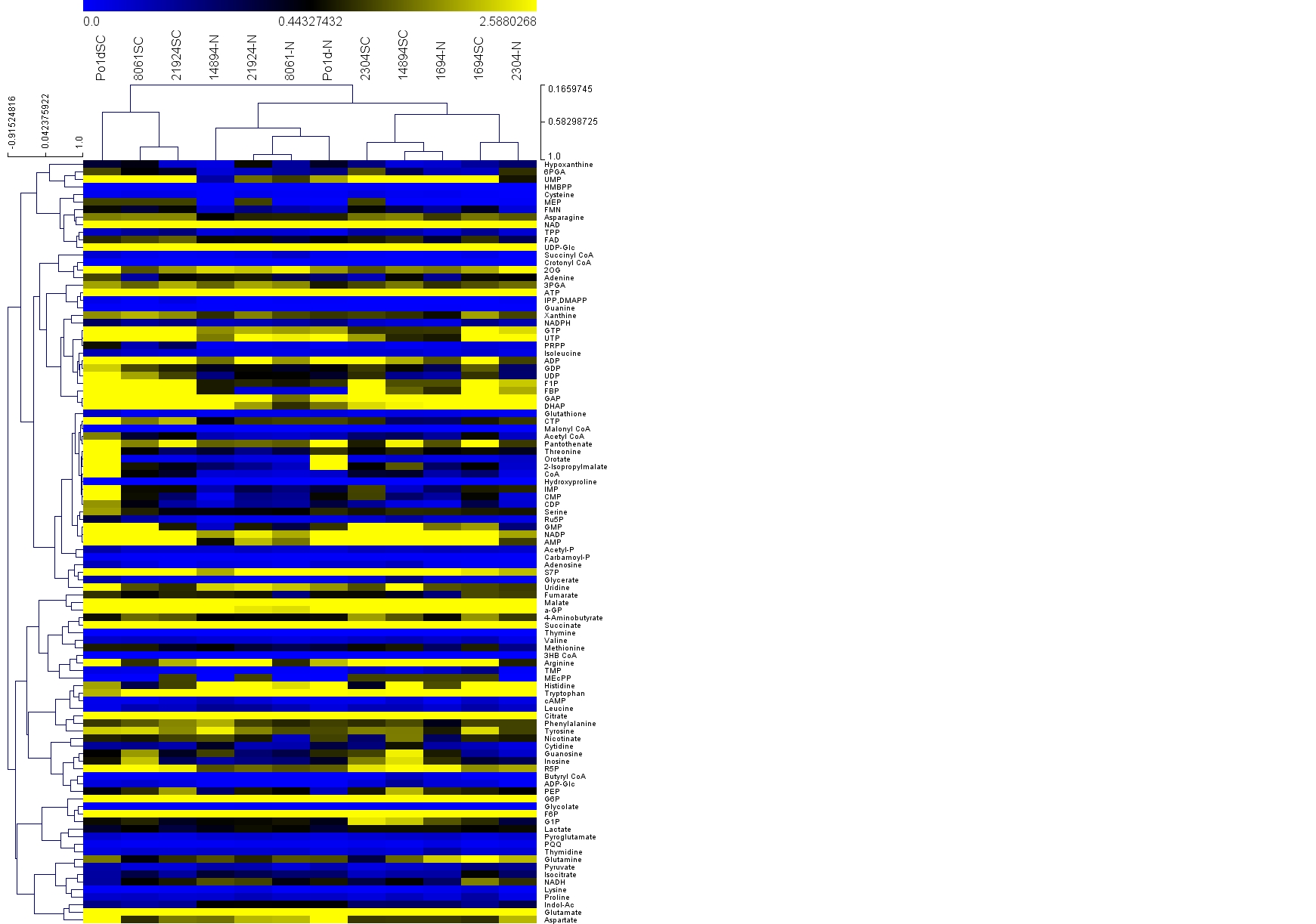

Supplement: Supplementary file 1 [file metabolites-11-00016-s001.zip › Figure S5.jpg]
